# Biodegradation of Emerging Pharmaceuticals from Domestic Wastewater by Membrane Bioreactor: The Effect of Solid Retention Time

**DOI:** 10.3390/ijerph18073395

**Published:** 2021-03-25

**Authors:** Raghad Asad Kadhim ALOBAIDI, Kubra ULUCAN-ALTUNTAS, Rasha Khalid Sabri MHEMID, Neslihan MANAV-DEMIR, Ozer CINAR

**Affiliations:** 1 Department of Environmental Engineering, Faculty of Civil Engineering, Yildiz Technical University, Istanbul 34220, Turkey; nmanav@yildiz.edu.tr (N.M.-D.); ocinar@yildiz.edu.tr (O.C.); 2 Ministry of Health and Environment, Baghdad, Iraq; 3 Department of Environmental Technologies, College of Environmental Science and Technologies, Mosul University, Mosul 41002, Iraq; rashamhemid@uomosul.edu.iq

**Keywords:** biodegradation, solid retention time, pharmaceuticals, membrane bioreactor, solid-phase extraction, by-products

## Abstract

Although conventional biological treatment plants can remove basic pollutants, they are ineffective at removing recalcitrant pollutants. Membrane bioreactors contain promising technology and have the advantages of better effluent quality and lower sludge production compared to those of conventional biological treatment processes. In this study, the removal of pharmaceutical compounds by membrane bioreactors under different solid retention times (SRTs) was investigated. To study the effect of SRT on the removal of emerging pharmaceuticals, the levels of pharmaceuticals were measured over 96 days for the following retention times: 20, 30, and 40-day SRT. It was found that the 40-day SRT had the optimum performance in terms of the pharmaceuticals’ elimination. The removal efficiencies of the chemical oxygen demand (COD) for each selected SRT were higher than 96% at steady-state conditions. The highest degradation efficiency was observed for paracetamol. Paracetamol was the most removed compound followed by ranitidine, atenolol, bezafibrate, diclofenac, and carbamazepine. The microbial community at the phylum level was also analyzed to understand the biodegradability of pharmaceuticals. It was noticed that the Proteobacteria phylum increased from 46.8% to 60.0% after 96 days with the pharmaceuticals. The Actinobacteria class, which can metabolize paracetamol, carbamazepine, and atenolol, was also increased from 9.1% to 17.9% after adding pharmaceuticals. The by-products of diclofenac, bezafibrate, and carbamazepine were observed in the effluent samples.

## 1. Introduction

The increase in the use of micropollutants, including pharmaceuticals, has become a major environmental problem in recent years. While the presence of pharmaceutical compounds is limited in the environment, knowledge of the long-term threats to aquatic species and human health even with low levels of drugs remains lacking. The concentration and effects of micropollutants on the environment and, perhaps, human beings have increased over the past few decades. Many micropollutants are persistent, only partially removed during treatment, and when disposed of in the environment over a long period, cause substantial pollution. The European directive, Water Framework Directive 2000/60/EC, is a global framework that underlines the importance of micropollutants in superficial water (rivers and lakes), transitional water (estuaries), coastal waters, and groundwater.

Although traditional water and wastewater treatment plants are successful in controlling pollutants such as organic substances and nutrients, they cannot remove the micropollutants (MPs). These micropollutants are substances found in low concentrations (ng/L and µg/L) in water and wastewater and consist of metals, hydrocarbons, surfactants, hormones, and pharmaceutical products. According to many studies, conventional wastewater treatment systems are insufficient to degrade certain pharmaceutical compounds, as a result, these compounds can be found in surface and groundwater [1,2,3]. In particular, pharmaceutical compounds are biologically active compounds that affect microorganisms upon their discharge to the aquatic environment. They are recalcitrant compounds that take a long time to break down in nature, therefore, effective methods of wastewater treatment that allow for the efficient removal of emerging pollutants at trace levels are increasingly needed. In wastewater treatment, MBR (membrane bioreactor) systems have been used more commonly recently because of their high effluent quality performance [4]. MBRs are effective in the treatment of many organic and inorganic pollutants, offering three basic advantages: (i) enhanced adsorption capacities through improved biomass characteristics; (ii) improved high sludge biodegradation, (iii) direct removal of several contaminants adsorbed on rejected particles through the membrane [5]. These process benefits are commonly recognized over the those of the traditional activated sludge process [6]; the most cited benefit is the decrease of sludge production resulting from operation at high solid retention times (SRTs). In general, hydrophobic and readily-biodegradable contaminants are very effectively eliminated by MBRs. However, hydrophilic compounds, in particular biodegradation-resistant ones, may not be excluded [7]. The substantial difference in MBR removal efficiency for several pharmaceuticals, from near-complete removal (e.g., paracetamol and bezafibrate) to almost no removal for some others (for example, carbamazepine and diclofenac) [8,9,10], has been noted previously.

Removal mechanisms do not follow a general rule as their relative contribution is dependent on the physicochemical properties of the micropollutant, its origin and composition, and the wastewater operational parameters [11]. A significant point is to understand why the removal of micropollutants such as pharmaceuticals by MBRs are affected by the treatment conditions applied in terms of the solid retention time (SRT). A single compound may vary in removal rate and this may be correlated with the physicochemical characteristics of the xenobiotics.

Various analytical determination methods of pharmaceutical compounds in wastewater are already available in the literature, which records specific pharmaceutical existence at levels from ng/L to g/L [12]. Multi-residual analytical methodologies, including pharmaceutical products of the multi-class range, are the tools required to provide a reliable and broad knowledge base on the presence of pharmaceuticals and for monitoring their removal, partitioning, and final fate. When analyzing several different physicochemical compounds simultaneously, a high recovery rate for each compound may not be seen. Therefore, a common experimental condition must be determined. However, the creation of the multicomponent analysis method is useful due to the reduced number of analyses that are required. The pre-concentration and isolation of the goal analytes can be performed by solid-phase extraction (SPE). The determination of the overall SPE method is important because most of these multicomponent analysis methods consist of two or more different sorbent materials and solvents used for elution, and they involve grouping target compounds according to their physicochemical properties.

Given these facts, the purpose of this work was the development of a sensitive multicomponent solid-phase extraction method to simultaneously analyze, by LC/MS-MS, six different therapeutic classified pharmaceuticals in synthetic domestic wastewater and to investigate the influences of SRT on treatment performance of the MBR in terms of removing these pharmaceuticals from synthetic domestic wastewater. The change in microbial species due to the micropollutants was also studied.

## 2. Materials and Methods

### 2.1. Design of the MBR System and Its Operation

The reactor was constructed according to the design and configuration for the size and dimensions of the membrane modules equipped with two PES (polyethersulfone) membranes with 0.45 µm and 0.20 µm pore sizes (Sterlitech Cooperation, Kent, WA, USA).

**Figure 1:**
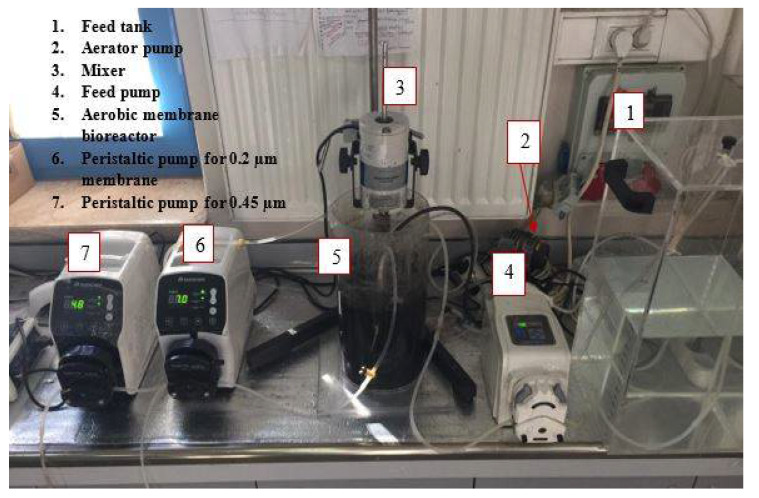
Lab-scale set up for submerged membrane bioreactor.

The aerobic membrane bioreactor was made of a plexiglass material with a cylindrical shape of 17.5 cm diameter corresponding to an 8 L volume and an active operating volume of 4 L. The peristaltic pumps were used to provide pressure for maintaining constant permeate flux and for automatic inoculation of the reactor feed (Shenchen Cooperation, Baoding, China). A level sensor was used to balance the water level of the reactor (Tin Muhendislik, Istanbul, Turkey). The reactor’s oxygen demand was provided by ambient air through the air pumps and diffusers. Details of the reactor configuration are illustrated in Figure 1.

The aerobic submerged membrane bioreactor (AeSMBR) was fed with synthetic domestic wastewater prepared according to the following recipe: 50 mg·L^−1^ NH_4_CI, 0.04 mg·L^−1^ MnCI_2_·4H_2_O, 0.132 mg·L^−1^ ZnCl_2_, 4.5 mg·L^−1^ K_2_HPO_4_, 4.2 mg·L^−1^ KH_2_PO_4_, 0.2 mg·L^−1^ Na_2_SO_3_·5H_2_O, 0.1 mg·L^−1^ CuCl_2_·2H_2_O, 2 mg·L^−1^ FeCl_3_·6H_2_O, 0.1 mg·L^−1^ NiCl_2_·6H_2_O, 0.03 mg·L^−1^ CoCl_2_·6H_2_O, 4.4 mg·L^−1^ CaCl_2_·2H_2_O, 12.2 mg·L^−1^ MgSO_4_·7H_2_O, and 40 mg·L^−1^ peptone. To obtain a 750 mg·L^−1^ concentration of chemical oxygen demand (COD), glucose was added. The membrane tank was equipped with two polyethersulfones (PES) submerged flat sheet microfiltration modules, one with a pore size of 0.45 µm and the other with a pore size 0.20 µm. Each membrane module comprised 12 × 12 cm plexiglass, withan active surface area of 56.25 cm^2^ (7.5 × 7.5 cm).

As defined in fluid mechanics, flux is the volume of a fluid passing through a unit area at a specific time. In this context, flux can be formulated as:

(1) $$J=\frac{\left(Volume\right)}{\left(Time\;\times \;Area\right)}$$

To provide adequate relaxation time for the membrane, the reactor was operated for 4.5 min and stopped for 30 s. In this respect, the flux value needed to be recalculated by considering the relaxation time so that the net flux calculations could be rearrange as below:

(2) $$Net\;Flux=\frac{Flux\;\times \;Running\;Time}{Waiting\;time+Running\;Time}=\frac{11.11\;\times \;4.5\;\min}{\left(0.5+4.5\right)\;\min}≅10\;\mathrm{LMH}$$

### 2.2. Samples Preparation

The target compounds were chosen because of their prevalence in the aquatic environment worldwide, ubiquity, and high human intake. Target compounds were categorized as shown in Table 1 according to their therapeutic effect and physicochemical properties.

All pharmaceutical standards used were of high purity grade (>98%). Diclofenac, acetaminophen, bezafibrate, carbamazepine, ranitidine hydrochloride, and atenolol were purchased from Alfa Aesar (Kandel, Germany). The method developed consisted of one extraction step for all target compounds, which greatly simplified sample preparation. Individual standard stock solutions in ethanol were prepared based on weight and stored at −2 °C. An acceptable dilution of individual stock solutions was established for a mixture of all pharmaceuticals.

According to the literature review [15,16,17,18], the concentrations of this study analytes was chosen as 12 µg/L carbamazepine, 20 µg/L diclofenac, 20 µg/L atenolol, 20 µg/L ranitidine hydrochloride, 12 µg/L bezafibrate, and 100 µg/L acetaminophen (paracetamol).

### 2.3. Solid-Phase Extraction Method 

Development, optimization, and validation of a method followed by liquid chromatography–triple quadrupole mass spectrometry (LC/MS-MS) were performed for the simultaneous determination of six multi-class pharmaceuticals using offline solid-phase extraction (SPE). The SPE method was inspired by the literature [19]. For conditioning applications, acetone (HPLC grade), methanol (HPLC grade), and ultrapure water were used (Merck, Istanbul, Turkey). Table 2 shows the conditions that were applied, including conditioning, percolation, washing, drying, and elution. 

To concentrate the samples, the compounds were extracted via the cartridges given in Table 2 using the SPE manifold system equipped with a vacuum pump. To optimize the extraction method, the performances of various SPE cartridge materials (Oasis HLB (200 mg, 6 mL) from Waters Corporation and C18 (500 mg, 6 mL) from Agilent) were compared. The detailed procedure is summarized in Table 2; firstly, SPE cartridges for samples 1 and 3 were conditioned with 5 mL MeOH followed by 5 mL distilled water at a flow rate of l mL/min. The same procedure was applied for the conditioning of SPE cartridges for samples 2 and 4 but it was proceeded with a conditioning of 5 mL of acetone. The water samples were percolated by the cartridges following the conditioning stage. The cartridge was then rinsed with 5 mL HPLC-grade water and dried for 15 min under a vacuum to extract the excess water. Elution of 2 × 4 mL of methanol was performed. For direct testing by LC/MS-MS analysis, the extract was evaporated under a gentle nitrogen stream and concentrated into 1 mL of sample volume. 

### 2.4. Analytical Methods

The feed wastewater characterization and reactor performance were monitored by wet analyses. For the suspended solid (TSS) parameter, standard methods (APHA, 1998) were applied. COD is one of the most important parameters in determining the degree of organic pollution in wastewaters. COD analyses were carried out using the microdigestion and titration method specified in the standard methods (SM 5220B) [20]. 

An HPLC (Shimadzu) equipped with a Diode-Array Detection (DAD) detector and LC-QTOF-MS/MS (Agilent 6530 Accurate Mass–ESI Interface, Santa Clara, CA, USA) was used for liquid chromatography analysis. To achieve the chromatographic separation, the Purospher Star RP-18 column (125 mm × 2.0 mm, particle size 5 µm) was supplied with a C18 guard column by Merck (Darmstadt, Germany). The analyses were performed in Positive Ionization mode with eluent A (acetonitrile-methanol (2:1)) and eluent B (ammonium acetate 5 mM at pH 4.7 (acetic acid)). The flow rate was selected as 0.3 mL/min, and the injection volume was determined as 10 µL. According to the selected method, the eluent gradient started from 5% and rose to 95% of eluent A in 5 min (was held for 4 min) and turned to the initial condition in 5 min.

### 2.5. Metagenomic Analysis

Biomass samples at the beginning of the steady-state of the 40-day SRT (before adding pharmaceuticals) and at the end of the 40-day SRT (after adding pharmaceuticals) in the aerobic membrane bioreactor were collected and stored immediately at −4 °C to evaluate the percentage of the microbial community. The samples were processed and analyzed as explained below.

### 2.6. DNA Isolation

The samples with an amount of 200 mg were transferred to tubes containing 0.1 mm diameter glass beads and 300 µL of lysis buffer (200 mM Tris-HCl, pH 8.0; 20 mM EDTA; 10% TritonX-100) and were homogenized for 1 min at 6000 rpm. The samples were transferred to new tubes to isolate the beads and were incubated at the 37 °C for 15 min; then, 10 μL of the lysozyme solution (200 μg/μL) was added. Next, 250 µL of lysis buffer (0.5 µg/µL Proteinase K, 5% Tween^®^ 20, 3M Guanidine thiocyanate, 20 mM Tris-HCl, pH 8.0) was added to each sample, and the samples were incubated for 15 min at 70 °C, followed by 5 min at 95 °C. After incubation, 250 µL of 2-propanol was appended to each tube and the samples were loaded onto the silica column. The DNA in the samples were passed through the silica column by centrifugation at 13,000 rpm for 1 min and kept by the silica column, then they were washed twice with wash solution (20 mM NaCl, 2 mM Tris-HCl, pH 8; 80% *v*/*v* ethanol). The silica column was dried by centrifugation. DNA retained in the silica columns was taken from the columns with 50 µL of 100 mM Tris-HCl prepared with nuclease-free, sterile, deionized water (pH 7) and preserved at −20 °C until analysis. Spectrophotometric methods measured the quantity and consistency of the DNA and checked its suitability for the following steps. Other molecular processes were performed using DNA with an OD260/OD280 ratio of 1.8–2.0, an OD260/OD230 ratio of 2.0–2.2, and at least ten ng/µL (preferably 50–300 ng/µL) concentration.

### 2.7. Next Generation Sequencing (NGS)

The primary pair was used to create amplicon libraries that covered about a 460 bp region and the V3-V4 region of the 16S rRNA gene [21]. Connector DNA sequences were added to the 5′ end of the target-specific primer pairs for compatibility with the Illumina index and sequence adapters of the generated library. The forward primer sequence of the primer-connector oligos specific for 16Sr RNA was 5′ TCGTCGGCAGCGTCAGATGT-GTATAAGAGACAGCCTACGGGNGGCWGCAG 3′, and the reverse primer sequence was 5′ GTCTCGTGGGCTCGGAGATGTGTATAAGAGACAGGACTACHVGGGTATCT AATCC-3′. The first PCR step was performed using “Bio-Speedy^®^ 2X qPCR Mix” and 200 nm from each primer.

The following thermal cycling program was monitored on the Biorad CFX Connect (Bio-Rad, Hercules, CA, USA): 3 min at 95 °C; 25 cycles of 30 s at 95 °C, 30 s at 55 °C, and 30 s at 72 °C; 5 min at 72 °C. By carrying out agarose gel electrophoresis of the PCR, the product size (~550 bp) was confirmed and “Bio-Speedy^®^ PCR Product Cleaning Kit” (Bioeks, Istanbul, Turkey) was used as an eluent.

By performing the second PCR step, binary index and Illumina sequencing adapters were included in the first PCR amplicons by using the Nextera XT Index Kit (Illumina, New York, NY, USA) then, the following program was used for thermal cycling: 95 °C for 3 min; 8 cycles of 30 s at 95 °C, 30 s at 55 °C, and 30 s at 72 °C; 5 min at 72 °C. PCR products were cleaned up with a “Bio-Speedy^®^ PCR Product Cleaning Kit” (Bioeks, Istanbul, Turkey). The final library was checked for size (~630 bp) by using the “Bioanalyzer DNA 1000 chip”. To form a library pool, the final library was diluted to 4 nM using 10 mM Tris pH 8.5 and 5 μL aliquots.

The collected libraries were denatured with NaOH, diluted with hybridization buffer (HT1), and denatured by temperature for batch forming and sequencing preparation, before the MiSeq sequencing. The research was performed using Illumina MiSeq v3 reaction kits. For each reaction, as an internal control, a minimum of 5% PhiX was added.

Mothur version 1.39.1 was used to examine the unprocessed sequence data (forward and reverse reads merged). The first step was to cut out the index and main sequences and then classify the particular sequences. The trimmed unique sequences were aligned using the RDP database sequences and the blastn algorithm. Filtering and error checking were done to unaligned sequences at both ends of the sequences. Pollution was prevented by pre-clustering. The UCHIME [22] code was used for the chimera removal. The sequences were categorized using the Bayesian classifier built into Mothur. With the support of the RDP database, reference and taxonomy files were gained. After the operational taxonomic unit (OTU) was selected and taxonomic determination was made according to the RDP database, OTUs were grouped according to their phylotypes.

## 3. Results

### 3.1. Determination of the SPE Method

**Figure 2:**
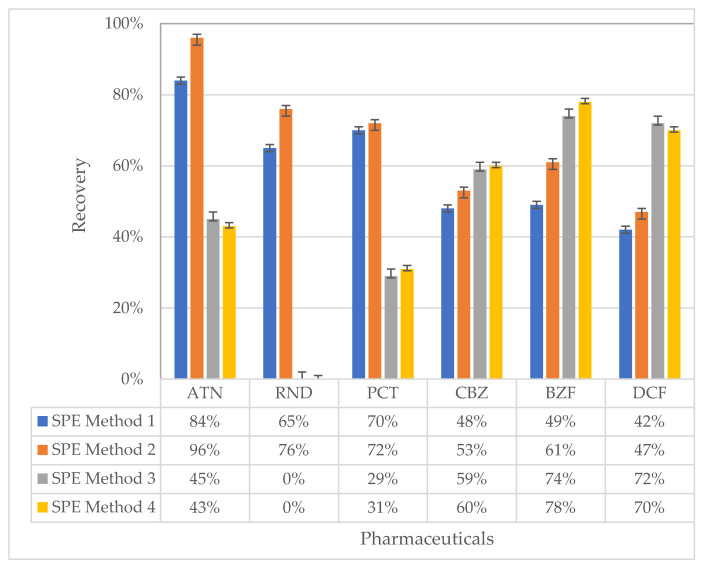
Recoveries for the extraction of selected pharmaceuticals in synthetic wastewater by using four different SPE methods.

To analyze the selected compound by LC/MS-MS, the selected compounds were required to be concentrated by the solid-phase extraction method: different solvents and cartridges were compared for their recoveries. The summarized methods are given in Table 2. The concentrations of atenolol, ranitidine, paracetamol, carbamazepine, bezafibrate, and diclofenac were 20, 20, 100, 12, 12, and, 20 µg/L, respectively. The volume of the samples used to analyze recoveries was 100 mL. The calculated recoveries can be seen in Figure 2.

While ranitidine can be extracted by HLB OASIS, no recoveries were observed by C18 cartridges. For atenolol, HLB OASIS (higher than 86%) gave better recoveries than did C18 cartridges (approximately 45%). Similarly, HLB OASIS was more efficient (70%) than C18 for paracetamol. Oasis HLB provided better results for atenolol, paracetamol, and ranitidine compared to those for Isolute C18, while Isolute C18 provided better results for carbamazepine, bezafibrate, and diclofenac compared to those of Oasis HLB. The recoveries were 60, 78, and 70% with the SPE method 4 for carbamazepine, bezafibrate, and diclofenac, respectively. Accordingly, SPE method 1 was chosen for the analysis of atenolol, ranitidine, and paracetamol, and SPE method 4 was chosen for the analysis of carbamazepine, bezafibrate, and diclofenac for all the analyses in this study.

### 3.2. Effect of SRT on COD Removal and MLSS Concentration

The bioreactor shown in Figure 1 was filled with aerobic activated sludge brought from the Atakoy Wastewater Treatment Plant in Istanbul. The reactor was operated at hydraulic retention time (HRT) (17.7 h), which corresponds to a permeate flux of 10 L/m^2^·h.

**Figure 3:**
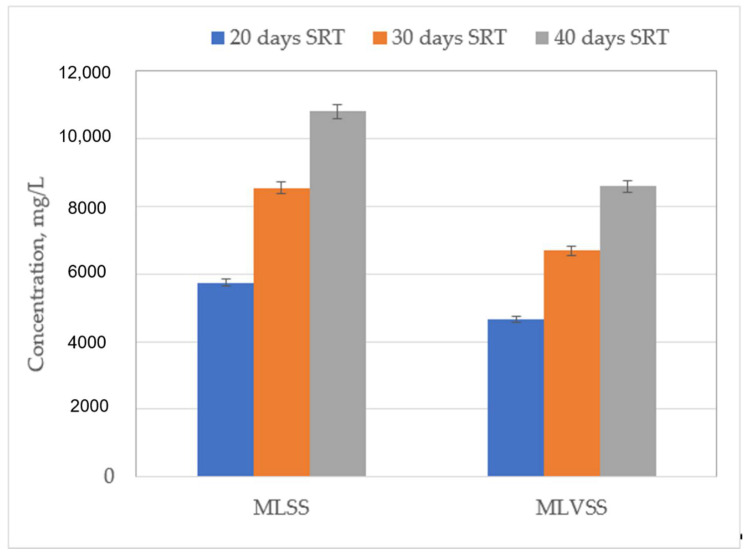
The average of MLSS and MLVSS concentrations in different solid retention times (SRTs).

**Figure 4:**
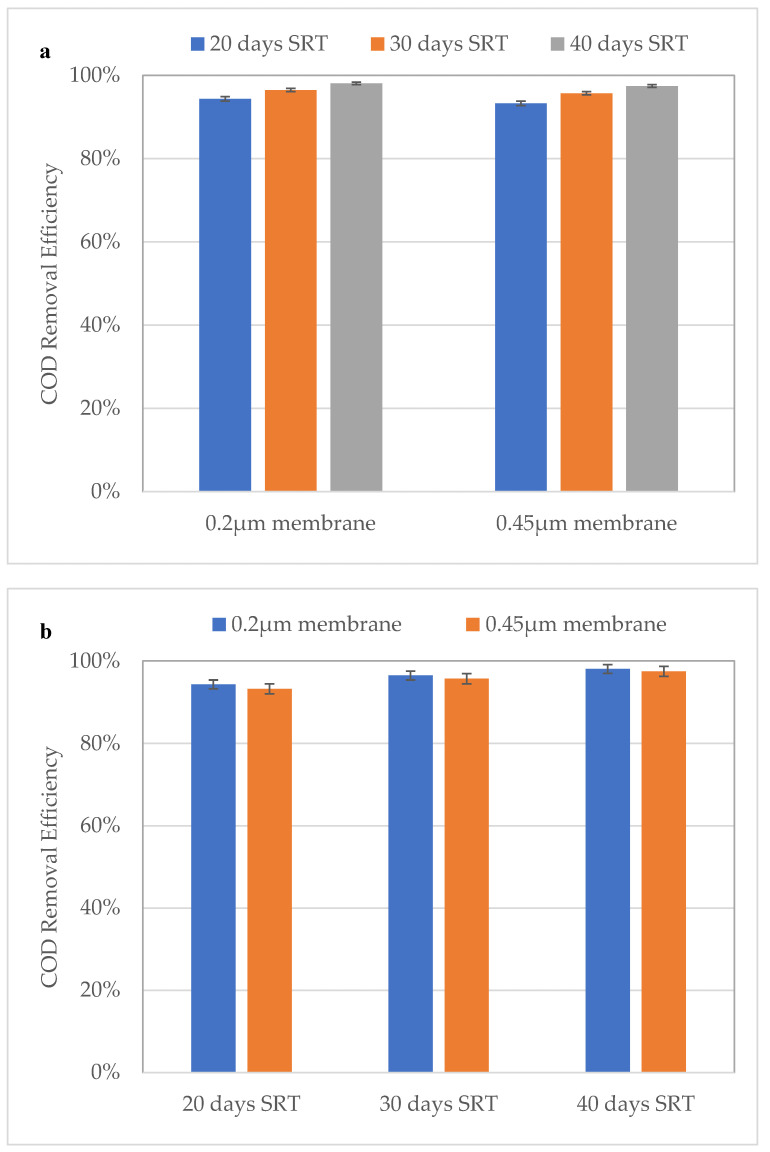
COD removal efficiency based on membrane type (**a**) and different SRTs (**b**).

During the time to reach the steady-state, the reactor was fed with synthetic domestic wastewater without MPs. After reaching a steady-state, the addition of MPs was started. All SRT studies were evaluated separately, and for each of them, the reactor was prepared from the starting point to reach steady-state conditions. To evaluate the effect of sludge retention time on COD degradation and bacterial growth, the reactor was controlled for 96 days after it reached steady-state conditions. The results of mixed liquor suspended solids (MLSS), mixed liquor volatile suspended solids (MLVSS) and, COD are seen in Figure 3 and Figure 4, respectively.

During the 20-day SRT, the COD removal efficiency for the 0.45 µm membrane was between 93.1 and 93.5%, and for 0.20 µm membrane it was 94.2–94.5% (Figure 4). In addition, the MLSS values ranged between 5492 and 5870 mg/L, and the MLVSS varied between 4514 and 4872 mg/L (Figure 3). Similarly, at 30 days of SRT, COD removal efficiencies increased slightly (95.6–95.9% for the 0.45 µm membrane and 96.3–96.7% for the 0.2 µm membrane), however, MLSS and MLVSS concentrations were 1.5 times higher than the results of the 20-day SRT. Conversely, the improvements in COD removal efficiencies were much higher and were 97.3–97.7% and 98.0–98.2% for the 0.45 and 0.2 µm membranes at 40-day SRT, respectively. The MLSS and MLVSS increased by approximately 2 times those of the 20-day SRT. These results are indeed comparable with the study of Broeck et al. (2012) who reported that increasing SRT increases the concentration of sludge solids and a longer SRT boosts COD removal treatment efficiency and produces less sludge [23].

Because of the high SRT values and complete solid retention inside the MBR, the biodiversity of the microorganisms is promoted and even slowly growing and free-living bacteria remain in the reactor, which leads to better performance [24].

### 3.3. Effect of SRT on the Removal of MPs

**Figure 5:**
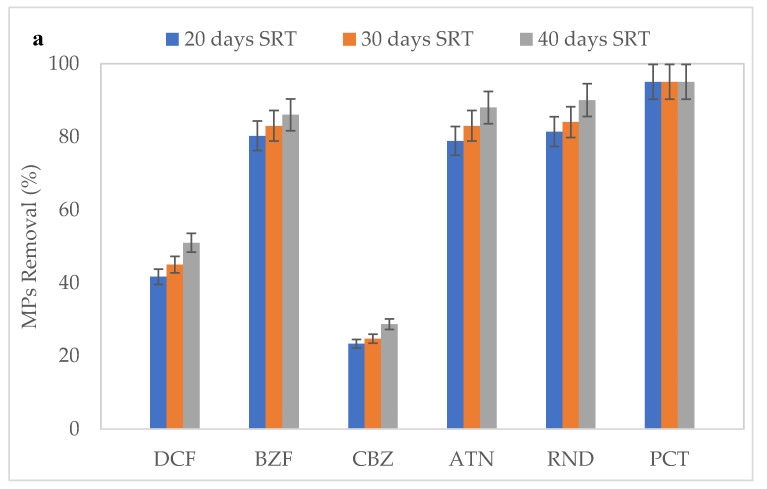

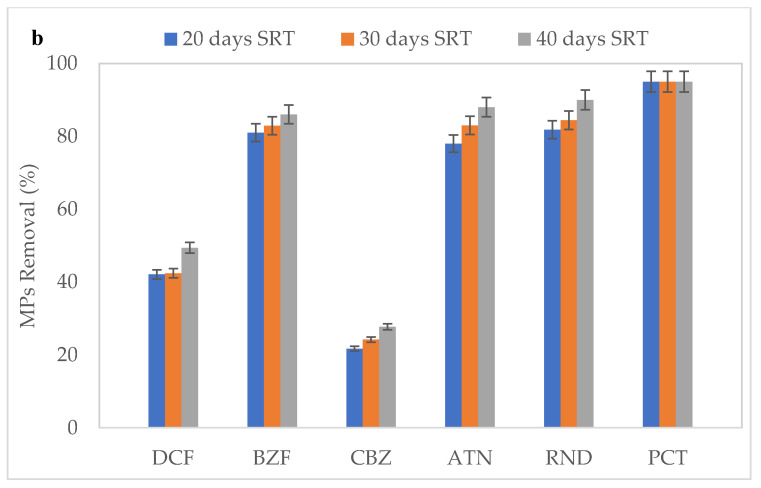
Micropollutants (MPs) removal under different SRTs in the membrane bioreactor (MBR) system equipped with (**a**) 0.2 µm and (**b**) 0.45 µm membranes (DCF: Diclofenac, BZF: Bezafibrate, CBZ: Carbamazepine, ATN: Atenolol, RND: Ranitidine, PCT: Paracetamol).

During the acclimatization phase, the reactor was fed with only synthetic domestic wastewater and when the reactor reached steady-state conditions, the mixture of atenolol, ranitidine, paracetamol, carbamazepine, bezafibrate, and diclofenac was pumped with a feed pump shown in Figure 1. The removal was monitored for 1 week and the samples were collected accordingly for 20, 30, and 40-day SRT. The results are given in Table 3 in detail, and in Figure 5 the removal efficiencies are shown for different SRTs passed through the 0.2 µm and 0.45 µm membranes-equipped MBR system. The increase in MLSS and MLVSS is recognized as one of the most important parameters promoting the biodegradation of pharmaceuticals and personal care products (PPCPs) [25]. According to these results, the 40-day SRT was selected as the best condition for the steady-state conditions. The concentrations, standard deviations, LOD, and LOQ of the studied compounds can be found in Table 3, and the comparison of the removal efficiencies of all compounds is given in Figure 5.

Paracetamol was detected as under the limit of detection at the 20, 30, and 40-day SRT, and according to LOD, paracetamol concentrations were obtained lower than 5.3 µg/L for all conditions. Even the highest concentration within all selected MPs was paracetamol (100 µg/L), the highest removal efficiency was obtained for paracetamol for all SRTs. The lowest removal efficiency was observed for carbamazepine for both membranes (approximately 28%) regardless of varying SRTs. This low removal efficiency was also observed by the authors of [25]. The diclofenac removal efficiency was observed at approximately 50%, which was similar to the study conducted by Mousel et al. (2021) [26] who studied the ultrafiltration membrane bioreactor (UF-MBR) process in the degradation of several pharmaceuticals from raw hospital wastewater and found that the removal of anti-inflammatory drugs (ibuprofen and paracetamol) was highly efficient. Paracetamol was the most removed compound followed by ranitidine, atenolol, bezafibrate, diclofenac, and carbamazepine for both membranes. These results align with many previous works [27,28]. It can be concluded that the MBR treatment with several SRT conditions can remove some of the MPs at high removal efficiencies, however, a further treatment process is required for total removal.

The concentration of all studied compounds was decreased with the increase of SRT. It can be observed in Figure 3 that MLSS showed an increase in concentration with the increase of SRT. The increase in MLSS should assist the sorption of selected pharmaceuticals [25,29]. While the increased SRT can generally boost the biological variety of slowly growing bacteria and longer sludge retention promotes the removal of MPs, some researchers have reported that diclofenac and carbamazepine removal is not affected significantly by SRT changes in MBR treatment [30].

### 3.4. Removal Mechanism and Possible Pathways

The pharmaceutical removal mechanisms in MBR treatment are complex and characterized by four main routes: (i) biotransformation or degradation; (ii) sorption by the sludge; (iii) volatilization or aeration striping; and, (iv) physical removal through membranes [30]. The constant of Henry’s Law for selected pharmaceuticals is lower than <10^−6^, which shows the removal of these compounds by volatilization is negligible (Table 1). Moreover, microfiltration (MF) membranes usually have a molecular weight cut off (MWCO) above several thousand daltons (Da), so that pharmaceutical retention (MWCO from 150 up to 350 Da) is insignificant in the MBR processes because of the exclusion of size. Consequently, biotransformation and sorption by sludge were presumably the dominating removing mechanisms.

For non-ionizable compounds, the hydrophobicity is described as log Kow, while it is described as log D for ionizable compounds; a compound is considered as hydrophobic when log D > 3.2 or log Kow > 2 [28]. The pKa values of bezafibrate and diclofenac are 3.61 and 4.15, respectively, (Table 1) so they are ionizable compounds, whereas the other pharmaceuticals showed no ionization. Accordingly, all the pharmaceuticals used in this study can be considered hydrophilic except for carbamazepine, which is a moderate hydrophobic compound.

The increased removal efficiency of hydrophobic pharmaceuticals can be due to (i) the dominant sorption by the sludge, which results in enhanced biological degradation, and (ii) having only electron-donating groups (EDGs) that improve oxidation [30]. The removal of carbamazepine is low in MBR processes because of the lack of an electron-donating group. Many other studies have shown that carbamazepine is not removed and recalcitrated in treatment with MBR due to poor degradability [5,8]. On the other hand, diclofenac was reduced at relatively low efficiency with median removal efficiencies of 49.4%, 42.4%, and 42.1% at SRTs of 40 days, 30 days, and 20 days, respectively. Other researchers have documented a slow or non-biotransformation rate of diclofenac [28,30]. The lower elimination rates of diclofenac and carbamazepine are due to the existence of multi-aromatic rings in their structure [11]. Paracetamol is highly degraded because its structure enables bacteria and enzymes to have unobstructed access to the exposed molecule that is later amended [31]. The removal of hydrophilic MPs ranged from low removal (diclofenac) to almost complete removal (paracetamol). The removal of hydrophilic MPs varies because MPs have different molecular structures and functional groups. Various removal efficiencies reported for hydrophilic MPs might be because (i) compounds having only EDGs could accomplish a high degree of removal; (ii) compounds with EDGs and e-withdrawing (EWGs) like amide and chloride in their molecular structure including paracetamol, diclofenac, and atenolol have diverse removal efficiencies; (iii) compounds that only have strong EWGs like carbamazepine showed low removal efficiency [30]. Therefore, the intrinsic biodegradability of these compounds has a great impact on the dominant removal mechanism of them, as the sludge sorption is less important.

The presence of possible by-products in the samples of the studies with an SRT period of 40 days and using a 0.2 µm membrane was detected using LC/-MS-MS. Accordingly, there were no by-products detected for paracetamol, atenolol, and ranitidine. The reason could be that the possible by-products could not be concentrated by the method used for SPE. The possible by-products determined for bezafibrate, carbamazepine, and diclofenac are listed in Table 4.

4-Chlorobenzoic acid was detected, which is the common metabolite for bezafibrate [32]. Furthermore, three possible by-products were determined for carbamazepine, which are named CBZ-TP1, CBZ-TP2, and CBZ-TP3, as given in Table 4. CBZ-TP1 could be formed by oxidation of the CBZ molecule (*m*/*z* = 253). Later CBZ-TP1 could form with the lost −CONH_2_ group and then form CBZ-TP2 and CBZ-TP3. The diclofenac compound also had some by-products that were observed in the activated sludge treatment processes. DCF-TP1 and DCF-TP2, which are DCF-lactam or 1-(2,6-dichlorophenyl)-1,3-dihydro-2H-indol-2-one and DCF-benzoic acid, were observed as given in Table 4 [33,34]. Microorganisms played a significant role in the transformation of carbamazepine, bezafibrate, and diclofenac by electrochemical reduction and oxidative transformation [35].

### 3.5. Bacterial Community Analysis

Inorganic and organic materials are oxidized by microorganisms in oxidation-reduction reactions to produce growth and maintenance energy. Under aerobic conditions, electron exchanges (food substrate for an organism) are often part of oxidation-reduction reactions and the electron acceptor is oxygen [38,39].

In this study, the 40-day SRT had the optimum performance in pharmaceuticals elimination and also in COD removal efficiency, so the metagenomic analysis was done for this period to understand the bacterial community before and after adding the pharmaceuticals.

**Figure 6:**
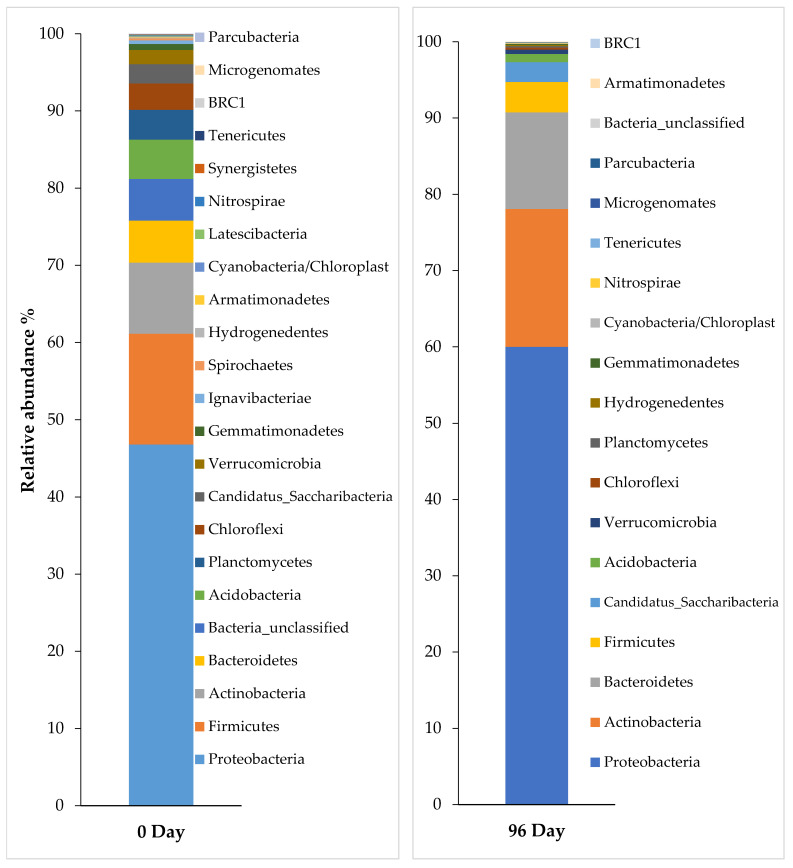
Profiles of bacterial community composition at the phylum level of activated sludge before (at 0 day—left) and after adding pharmaceuticals (at 96 day—right) in 40-day SRT.

**Figure 7:**
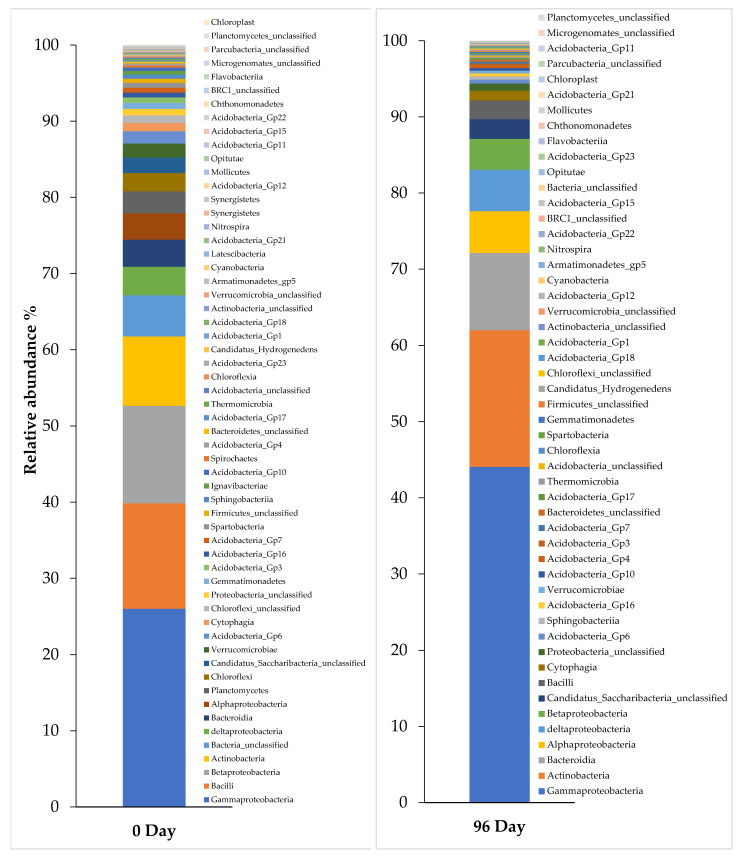
Profiles of bacterial community composition at the class level of activated sludge before (at 0 day—left) and after adding pharmaceuticals (at 96 day—right) in 40-day SRT.

Figure 6 shows the percentage of the microbial community at the phylum level inoculated in the aerobic MBR system of activated sludge, and the microbial community percentage for the same conditions at the class level is shown in Figure 7 at 0 days during the steady-state, which is the time before adding the pharmaceuticals, and also 96 days after adding the pharmaceuticals. As a result of the metagenomic analysis applied to this sample, a total of 23 phyla and 58 classes were identified. Based on 16S rRNA NGS data, the percentage of dominant microbial distributions in raw sludge were found to be 46.8% Proteobacteria, 14.3% Firmicutes, 9.2% Actinobacteria, and 5.4% Bacteroidetes. Proteobacteria represents the predominate heterotrophic bacterial phylum with Gammaproteobacteria, Betaproteobacteria, Deltaproteobacteria, and Alphaproteobacteria classes, the genus of these bacteria were identified to use paracetamol as the sole carbon and energy source [40]. It can be noticed that the Proteobacteria phylum was increased to 60.0% three months after adding the pharmaceuticals (Figure 5) and the Gammaproteobacteria class increased from 26% to 44% (Figure 6). The Actinobacteria class can metabolize paracetamol, carbamazepine, and atenolol, and it was also increased from 9.1% to 17.9% after adding pharmaceuticals [40]. 

In general, the key mechanism for the removal of pharmaceuticals by activated sludge is biodegradation. However, the elimination of environmental contaminants is not always successful due to the lack of degraders in the environment. Biological acclimation and bioaugmentation will overcome all these limitations [41]. Pure cultures and mixed cultures, which can remove the pollutants, can be dominated by biological acclimation and bioaugmentation in the biological treatment process. Pure cultures can be used to remove commonly detected carbamazepine, isolating it from the activated sludge, wastewater, or sediment. Pure carbamazepine has a stable structure leading to low biodegradability, however, two pure cultures will degrade carbamazepine in the presence of glucose: unidentified Basidiomycete [42], and Streptomyces MIUG [42,43]. White rot fungus Phanerochaete Sordida YK-624 can remove diclofenac entirely and in the absence of extra substrate can eliminate its lethal toxicity to organisms [41,42,43].

## 4. Conclusions

The biodegradation of six pharmaceuticals was investigated in a membrane bioreactor. After the reactor reached the steady-state condition, the degradation efficiencies of six targeted pharmaceuticals were determined under different sludge retention times. The highest degradation efficiencies were observed for paracetamol for each SRT followed by paracetamol, ranitidine, atenolol, bezafibrate, diclofenac, and carbamazepine. The bacterial community analysis was evaluated for the sludge in steady-state conditions and also after adding the pharmaceuticals. It was observed that Proteobacteria in the sludges increased from 46.8% to 60% while Actinobacteria increased from 9.1% to 17.9%. This reveals that microorganisms can metabolize the targeted pharmaceuticals. To understand the degradation mechanism, possible by-products were observed in the effluent samples, where the first-step degradation by-products of diclofenac, bezafibrate, and carbamazepine were detected. As a further study, the ratio of sludge adsorption and degradation of targeted pharmaceuticals can be studied to better understand whether the sludge requires additional treatment. Since the degradation of diclofenac and carbamazepine was low and they need to be degraded to reach the non-cancerogenic limits, the development of highly effective biodegradation of these compounds should also be a future focus.

## Figures and Tables

**Table 1:** Main characteristics of the selected and analyzed pharmaceuticals [13,14].

	Carbamazepine	Acetaminophen (Paracetamol)	Diclofenac (Sodium Salt)	Bezafibrate	Ranitidine Hydrochloride	Atenolol
Applications	Antiepileptic	Therapeutic	Anti-inflammatory	Lipid regulator	Anti-histamine	β-Blockers
CAS Number	298-46-4	103-90-2	15307-86-5	41859-67-0	66357-59-3	29122-68-7
Formula	C_15_H_12_N_2_O	C_8_H_9_NO_2_	C_14_H_10_Cl_2_NNaO_2_	C_19_H_20_ClNO_4_	C_13_H_22_N_4_O_3_S·HCl	C_14_H_22_N_2_O_3_
Log K_ow_	2.45	0.46	4.51	4.25	0.27	0.16
pK_a_	13.9	9.4	4.15	3.61	8	9.6
Log D	1.89	0.47	1.77	−0.93	−0.63	−2.09
Henry Constant (atm-m^3^/mole)	1.1 × 10^−12^	6.42 × 10^−13^	4.7×10^−12^	2.12 × 10^−15^	3.42 × 10^−15^	1.37 × 10^−18^
M.W.	236.27	151.17	318.14	361.82	350.86	266.34
Molecular Structure	* 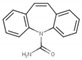 *	* 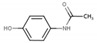 *	* 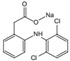 *	* 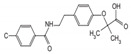 *	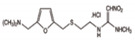	* 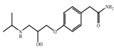 *

**Table 2:** Solid-phase extraction (SPE) method determination.

Applications	SPE Method 1	SPE Method 2	SPE Method 3	SPE Method 4
**Types of cartridge**	Oasis HLB cartridge 200 mg, 6 mL	Oasis HLB cartridge 200 mg, 6 mL	C18 cartridge, 500 mg, 6 mL	C18 cartridge, 500 mg, 6 mL
**Conditioning**	5 mL MeOH, 5 mL water	5 mL acetone, 5 mL MeOH, 5 mL water	5 mL MeOH, 5 mL water	5 mL acetone, 5 mL MeOH, 5 mL water
**Percolation**	100 mL	100 mL	100 mL	100 mL
**Washing**	5 mL water	5 mL water	5 mL water	5 mL water
**Drying**	15 min.	15 min.	15 min.	15 min.
**Elution**	8 mL MeOH	8 mL MeOH	8 mL MeOH	8 mL MeOH

**Table 3:** The concentrations of pharmaceuticals before and after biodegradation.

				40-Day SRT							30-Day SRT							20-Day SRT						
Comp.	R^2^	LOD	LOQ	Inf.	0.2 µm mem. MBR Eff.	RSD% (*n* = 3)	R.E. %	0.45 µm mem. MBR Eff.	RSD % (*n* = 3)	R.E.%	Inf.	0.2 µm mem. MBR Eff.	RSD % (*n* = 3)	R.E.%	0.45 µm mem. MBR Eff.	RSD % (*n* = 3)	R.E%	Inf.	0.2 µm mem. MBR Eff.	RSD % (*n* = 3)	R.E%	0.45 µm mem. MBR Eff.	RSD % (*n* = 3)	R.E%
DCF	0.997	1.3	4.39	20.76 ± 0.11	10.173 ± 0.19	1.875	51	10.49 ± 0.38	3.713	49.4	20.48 ± 0.34	11.23 ± 0.17	1.496	45	11.8 ± 0.16	1.4	42.4	20.82 ± 0.23	12.13 ± 0.16	1.281	41.7	12.05 ± 0.134	1.112	42.1
BZF	0.997	0.134	0.445	12.48 ± 0.21	1.72 ± 0.01	0.723	86	1.75 ± 0.02	1.4	86	12.44 ± 0.18	2.093 ± 0.01	0.225	83	2.126 ± 0.038	1.814	82.9	12.31 ± 0.15	2.43 ± 0.01	0.455	80.26	2.34 ± 0.025	0.752	81
CBZ	0.991	1.17	3.9	12.583 ± 0.15	8.96 ± 0.1	1.112	28.7	9.09 ± 0.08	0.933	27.7	12.693 ± 0.15	9.55 ± 0.15	1.539	24.7	9.62 ± 0.237	2.466	24.2	12.428 ± 0.17	9.529 ± 0.09	0.891	23.32	9.731 ± 0.067	0.688	21.7
ATN	0.992	0.64	2.13	20.81 ± 0.12	2.41 ± 0.04	1.79	88	2.51 ± 0.06	2.28	88	20.87 ± 0.04	3.52 ± 0.03	0.877	83	3.5 ± 0.054	1.55	83	20.81 ± 0.26	4.385 ± 0.02	0.522	78.9	4.58 ± 0.037	0.814	78
RND	0.997	0.665	2.22	20.48 ± 0.07	2.02 ± 0.03	1.4	90	2.028 ± 0.05	2.43	90	20.6 ± 0.27	3.28 ± 0.03	0.995	84	3.21 ± 0.088	2.77	84.4	20.52 ± 0.18	3.81 ± 0.01	0.228	81.4	3.73 ± 0.012	0.342	81.8
PCT	0.999	5.3	17.7	103.96 ± 0.81	n.d		≥95	n.d		≥95	104.033 ± 1.95	n.d		≥95	n.d		≥95	103.247 ± 1.26	n.d		≥95	n.d		≥95

DCF: Diclofenac, BZF: Bezafibrate, CBZ: Carbamazepine, ATN: Atenolol, RND: Ranitidine, PCT: Paracetamol, Inf: Influent, Eff. Effluent, LOD: Limit of Detection, LOQ: Limit of Quantification, RSD: Relative Standard Deviation, R.E.: Removal Efficiency. n.d: not detected.

**Table 4:** The possible by-products of diclofenac, bezafibrate, and carbamazepine that were detected [32,33,34,35,36,37].

PPCPs	Transformation Products	*m*/*z*	Structure	Formula
Diclofenac	DCF-TP1 DCF-lactam or 1-(2,6-dichlorophenyl)-1,3-dihydro-2H-indol-2-one	280.095	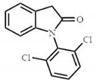	C_14_H_10_NOCl_2_
	DCF-TP2 DCF-benzoic acid	283.036	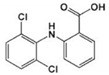	C_13_H_9_Cl_2_NO_2_
Bezafibrate	4-Chlorobenzoic acid	113.05	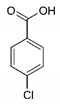	C_7_H_5_ClO_2_
Carbamazepine	CBZ-TP1	253.097	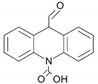	C_15_H_12_N_2_O_2_
	CBZ-TP2	210.015	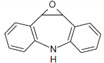	C_14_H_11_NO
	CBZ-TP3	224.125	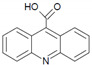	C_14_H_9_NO_2_

## Data Availability

All data are included in the article. Data sharing is not applicable to this article.
